# Bravo

**Published:** 1885-12

**Authors:** 


BRAVO.—Dr. P. H. Thompson, of Georgia, reports three cases of horrible monstrosity in the St. Louis *Courier of Medicine*, born of healthy mothers, all three of whom declare they had not seen, nor been frightened by any object calculated to make what are popularly supposed to be "maternal impressions on the fœtus in utero." The doctor comes to the defense of the women, and says that deformities of the kind are never produced by the emotions of the mother; that the mother really has nothing to do with it, she furnishing only the food for the germ of life, which is contributed by the male. He compares the female ovum to that of a hen, which, he says, is only so much nutritious food for the embryo to feed upon, and argues if there is any defect in the germ, if the male is crippled, disabled, or if the germ with which the ovum is fertilized, is imperfect—it cannot result in a perfect development; and the female should not be blamed for the result, as she only nurtures the seed planted in soil furnished by her. The doctor thus turns the tables on the "lords of creation," and blames them for the occurrence of monstrosities. There is much truth and justice in what he says, and the mothers of the land should return him a vote of thanks.

